# Disturbances across whole brain networks during reward anticipation in an abstinent addiction population

**DOI:** 10.1016/j.nicl.2020.102297

**Published:** 2020-05-26

**Authors:** Liam J. Nestor, John Suckling, Karen D. Ersche, Anna Murphy, John McGonigle, Csaba Orban, Louise M. Paterson, Laurence Reed, Eleanor Taylor, Remy Flechais, Dana Smith, Edward T. Bullmore, Rebecca Elliott, Bill Deakin, Ilan Rabiner, Anne-Lingford Hughes, Barbara J. Sahakian, Trevor W. Robbins, David J. Nutt, ICCAM Consortium

**Affiliations:** a Neuropsychopharmacology Unit, Centre for Psychiatry, Imperial College London, United Kingdom; b Department of Psychiatry, University of Cambridge, United Kingdom; d Department of Psychology, University of Cambridge, United Kingdom; c Neuroscience and Psychiatry Unit, University of Manchester, United Kingdom; e Centre for Neuroimaging Sciences, Institute of Psychiatry, Psychology and Neuroscience, King's College London, London, United Kingdom

## Abstract

The prevalent spatial distribution of abnormalities reported in cognitive fMRI studies in addiction suggests there are extensive disruptions across whole brain networks. Studies using resting state have reported disruptions in network connectivity in addiction, but these studies have not revealed characteristics of network functioning during critical psychological processes that are disrupted in addiction populations. Analytic methods that can capture key features of whole brain networks during psychological processes may be more sensitive in revealing additional and widespread neural disturbances in addiction, that are the provisions for relapse risk, and targets for medication development. The current study compared a substance addiction (ADD; n = 83) group in extended abstinence with a control (CON; n = 68) group on functional MRI (voxel-wise activation) and global network (connectivity) measures related to reward anticipation on a monetary incentive delay task. In the absence of group differences on MID performance, the ADD group showed reduced activation predominantly across temporal and visual regions, but not across the striatum. The ADD group also showed disruptions in global network connectivity (lower clustering coefficient and higher characteristic path length), and significantly less connectivity across a sub-network comprising frontal, temporal, limbic and striatal nodes. These results show that an addiction group in extended abstinence exhibit localised disruptions in brain activation, but more extensive disturbances in functional connectivity across whole brain networks. We propose that measures of global network functioning may be *more* sensitive in highlighting latent and more widespread neural disruptions during critical psychological processes in addiction and other psychiatric disorders.

## Highlights

• Analytical methods can capture key features of whole brain networks in addiction.
• We compared reward network connectivity in addiction (ADD) and control (CON) groups.
• The ADD group showed disruptions in global network connectivity.
• Global network measures may be *more* sensitive than traditional voxel-wise analyses.

## Graphical abstract

Network based statistics (NBS) analyses detected a graph sub-network comprising 153 edges between 59 nodes of the connectome where the ADD group demonstrated significantly less connectivity compared with the CON group. These differences in connectivity were mostly intra-hemispheric (55%), the majority (38%) being in the right hemisphere. The anatomical distribution of these connectivity differences between the two groups involved frontal (insula, inferior frontal gyrus, orbitofrontal cortex), limbic-associated (anterior cingulate gyrus, thalamus), and striatal (accumbens, caudate, pallidum) regions. The connectivity differences reported in this ADD sample indicate alterations between cognitive, striatal and limbic-associated regions during reward anticipation that persist into extended abstinence.

**Figure:**
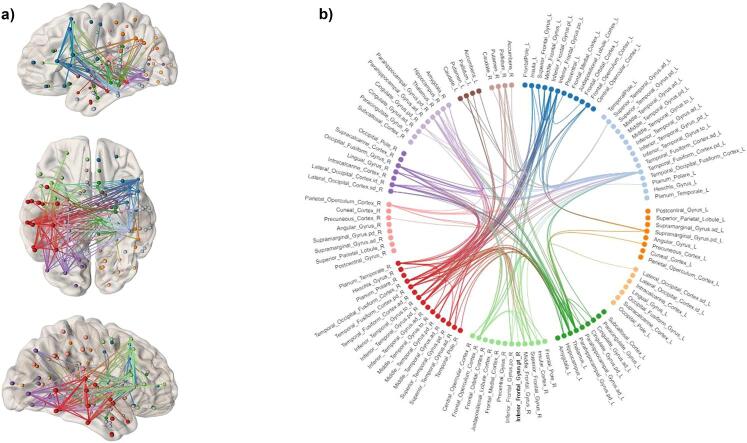

## 1 Introduction

Reward processing is a psychological construct that has evolved to drive incentive-based learning and the development of goal-directed behaviours in humans. Reward processing is mediated by a collection of subcortical and prefrontal cortical regions (Haber and Knutson, 2010, Knutson et al., 2001, O'Doherty et al., 2001) that are connected to form a complex neural network encoding various types of rewarding stimuli (Belin and Everitt, 2008, Draganski et al., 2008, Haber and Knutson, 2010). Substance addiction disorders are associated with disturbances within this reward network during the processing of non-drug rewards (Balodis and Potenza, 2015, Hommer et al., 2011, Just et al., 2019, Koob and Le Moal, 2005, Luijten et al., 2017, Wrase et al., 2007), and which are associated with drug relapse during abstinence (Gowin et al., 2015, Stewart et al., 2014). Most studies in addiction populations, however, only probe regional differences in brain functioning, and do not attempt to elucidate features of global connectivity across networks that respond during certain psychological processes, such as reward. Differences in global network connectivity likely underlie the regional differences commonly reported in functional MRI studies in addiction disorders, and therefore, may be more sensitive in revealing widespread neural disturbances in addiction disorders.

Connectivity across brain networks can be probed by examining various characteristics that relate to their topology and functioning (Bullmore and Sporns, 2009). These methods have been used to capture brain network connectivity during resting state in psychiatric populations (Baek et al., 2017, Bassett et al., 2008, Luo et al., 2015, Ye et al., 2015), including those with addiction disorders (Jiang et al., 2013, Morris et al., 2018, Sjoerds et al., 2017, Tschernegg et al., 2013, Yuan et al., 2010). These studies, however, have not been able to examine the characteristics of network functioning during critical psychological processes known to be disrupted in these populations. Studies examining connectivity during the psychological processes of cognitive control (Fornito et al., 2011, Ray et al., 2017) and reward (Manelis et al., 2016, Nestor et al., 2019, Verdejo-Roman et al., 2017), have been conducted, providing more precise measures of connectivity across networks that relate to these domains. Analytic methods that can reveal key features of brain network functioning during psychological processes may capture disruptions in connectivity that harbour the provisions for relapse risk in addiction disorders, and which may be clinical targets for medication development.

We have previously shown that abstinent substance-dependent populations demonstrate regional disturbances during reward processing (Murphy et al., 2017, Nestor et al., 2017), that likely represent disruptions in connectivity across global brain networks. This report documents a novel analytical approach that has attempted to identify differences in reward-related global network connectivity in an addiction population, an approach that departs from merely attempting to identify regional disturbances using common voxel-wise analyses. Here an addiction group in extended abstinence was compared with a control group on measures of voxel-wise *and* global network connectivity associated with reward anticipation on a monetary incentive delay task. We hypothesized that the addiction group would exhibit whole brain activation and global network connectivity disturbances, but that measures of connectivity would prove *more* sensitive to highlighting neural disturbances during reward processing in addiction.

## 2 Material and methods

### 2.1 Participants

Sixty-eight control (CON: mean age 39.79 ± 1.22; 18 females, 50 males) and 83 addiction (ADD: mean age 40.05 ± 0.92; 16 females, 67 males) participants completed the current study. The current dataset was collected as part of a multi-centre study involving three study sites in the United Kingdom (Imperial College London, University of Cambridge and University of Manchester – ICCAM). For a more detailed description of the ICCAM Platform, see Paterson et al. (2015) and McGonigle et al. (2017). Inclusion criteria were individuals who met DSM-IV measures for current or prior substance dependence (e.g., alcohol, cocaine, opiates). The ADD group consisted of 29 (35%) pure alcohol-dependent, 42 (50%) poly substance-dependent (e.g., alcohol and cocaine, cocaine and opiate) and 12 (15%) mono substance-dependent (e.g., cocaine, opiates) volunteers. While addiction studies typically recruit volunteers in early abstinence, there was no upper limit in the current study. The mean abstinence length from alcohol in the current sample was 15.00 ± 3.50 months, while for cocaine and opiates it was 27.99 ± 3.72 and 39.04 ± 7.75 months, respectively. Therefore, the current ADD group was made of a heterogenous sample with former substance-dependencies, and with variable levels of substance abstinence at the time of testing. This meant that there was no substance dependence measure that was shared by all members of the ADD group. The CON group had no previous history of substance abuse, as assessed using the Alcohol, Smoking and Substance Involvement Screening Test (ASSIST) (Group, 2002) and timeline follow-back. All participants were required to provide a negative breath alcohol test and a negative urine sample for various drugs of abuse on the day of testing (screening for the presence of amphetamines, benzodiazepines, cannabinoids, cocaine and opiates). The Mini*-*International Neuropsychiatric Interview *(*MINI*)* (Sheehan et al., 1998) was administered to all participants by a trained psychiatrist to screen for the presence of Axis I psychiatric disorders that were part of the study exclusion criteria.

Exclusion criteria included 1) current use of regular prescription or non-prescription medication that could not be stopped; 2) current primary axis I diagnosis, past history of psychosis (unless drug-induced); 3) current or past history of enduring severe mental illness (e.g., schizophrenia, bipolar affective disorder); 4) other current or past psychiatric history that, in the opinion of a psychiatrist, contraindicated participation; 5) history or presence of a significant neurological diagnosis that may have influenced the outcome or analysis of the results; 6) claustrophobia or unable to lie still in the MRI scanner for up to 90 min and 7) presence of a cardiac pacemaker, other electronic device or other MRI contraindication, including pregnancy, as assessed by a standard pre-MRI questionnaire. Secondary or lifetime history of depression or anxiety was permitted in both the ADD and CON groups since these are very common psychiatric disorders.

All participants provided written informed consent. The study was conducted in accordance with the Declaration of Helsinki. Ethical approval was obtained from West London and Gene Therapy Advisory Committee National Research Ethics Service Committee (11/H0707/9) and relevant research governance and Participant Identification Centre (PIC) approvals obtained.

## 3 Monetary incentive delay task (MID)

We used a “monetary incentive delay task” (MID), which was based on that originally employed by Knutson (Knutson et al., 2001), and which we have used to already publish data from the ICCAM platform (Murphy et al., 2017, Nestor et al., 2017). At the beginning of each trial, participants viewed one of three symbols (a cue) that indicated the potential to gain fifty pence (square containing an ascending arrow), lose fifty pence (square containing a descending arrow) or experience no financial outcome (square containing a horizontal line - here referred to as a neutral trial). Each cue was presented for one second, with a variable duration (2–4 s) for the subsequent anticipation period. Following the anticipation period, participants made a button press response upon the presentation of a visual target (star located within a circle). Following their response to the visual target, participants received feedback (1.5 s) as to whether they were successful (“Hit”) or unsuccessful (“Miss”) on that trial and saw a running total of their winnings up to that point in the task. Following feedback, there was an end fixation period (3–5 s) before the commencement of the next trial. Because the primary objective of ICCAM was to examine the neural correlates of reward anticipation, we chose to use a smaller number of loss trials in order to amplify the incentive salience of the gain trials during the task. Consequently, there was a total of 18 gain, 6 lose and 18 neutral trials on each run of the task. The MID task was tailored to adapt to the visual target reaction time of each participant by using a staircase algorithm, such that the presentation of the visual target became shorter as performance improved during the experiment. This enabled us to set a limit on the success rate of each participant (∼66%), which additionally served to incentivize participants to engage in the task. Participants were instructed to maximize their winnings and were told they would receive them at the end of the study. Dependent measures were accuracy (percentage) and mean reaction time (milliseconds) to the visual target on each of the gain, lose and neutral trials, and the amount won (£) on the task. Participants completed two runs of the task (432 s each) during scanning. The task was programmed using E-Prime version 2.0 (Psychology Software Tools, Pittsburgh, USA).

## 4 Statistics

Group demographics were compared using simple independent samples *t*-test analyses. For analyses conducted on MID performance, two (Group: CON vs. ADD) by two (Condition: Gain vs. Neutral) analyses of variance were conducted. The CON and ADD groups were also compared on the lose accuracy and lose reaction time performance measures, as well as the amount of money won (£), using analyses of variance. These analyses were conducted controlling for study site. For the graph measures (see below) we conducted two (Group: CON vs. ADD) by five (1 ⩽ K ⩽ 5) analyses of variance, while also controlling for study site. All these analyses were conducted using permutation testing (5000 iterations) in the R statistical software package (www.R-project.org).

## 5 Functional MRI (fMRI) Data acquisition

The ICCAM platform was designed to allow the rapid testing across sites of multiple compounds relevant to addiction treatment. Imaging at multiple sites in parallel on the ICCAM platform accelerated study completion, through the sharing of expertise, infrastructure and capacity. For a more comprehensive description of data acquisition across the three sites on ICCAM, please see McGonigle et al (McGonigle et al., 2017). Briefly, all centres operated MRI machines with a main magnetic field of 3 T (T). Centres in London and Cambridge operated nominally identical 3 T Siemens Tim Trio systems running the syngo MR B17 software with a Siemens 32 channel receive-only phased-array head coil. The Manchester centre operated a 3 T Philips Achieva running version 2.6.3.5 software and an 8 element SENSE head coil. For anatomical images, 160 high-resolution T1-weighted anatomic MPRAGE axial images (FOV 256 mm, thickness 1.0 mm, voxel size 1.0 × 1.0 × 1.0) were acquired (total duration 303 s). Functional data were acquired using a T2* weighted echo-planar imaging sequence collecting 36 non-contiguous (0% gap) 3.0 mm axial slices covering the entire brain (TE = 31 ms, TR = 2000 ms, FOV 225 mm, 64 × 64 mm matrix size in Fourier space). Each run of the MID task produced a total of 216 volumes of functional MRI data.

## 6 fMRI Data analyses

Data pre-processing and statistical analysis were conducted using FEAT (fMRI Expert Analysis Tool) from the FMRIB Software Library (www.fmrib.ox.ac.uk/fsl). Pre-statistical processing was as follows: motion correction utilizing FMRIB’s Linear Image Registration Tool (MCFLIRT; non-brain matter removal using Brain Extraction Tool (BET); spatial smoothing with a 5-mm full-width half maximum Gaussian kernel; mean-based intensity normalization; nonlinear high-pass temporal filtering (Gaussian-weighted least squares straight line fit, with sigma = 25.0 s). The six rigid body movement parameters were also included as regressors in the model in FSL FEAT.

For each participant, first level whole-brain mixed-effects analyses were performed by modelling the MID anticipation periods (i.e. gain, neutral) as explanatory variables within the context of the general linear model on a voxel-by-voxel basis (variable boxcar functions for the cue + variable anticipation period regressors were convolved with the haemodynamic response function). The gain and neutral outcome periods (“Hit” and “Miss”) were regressed out of the functional time series as conditions of no interest for the analyses reported here. During these first level analyses, the *gain anticipation > neutral* anticipation contrast was formulated. Owing to the small number of loss trials in the current task, the lose cue + anticipation and outcome periods (“Hit” and “Miss”) were additionally regressed out of the functional time series as conditions of no interest. Therefore, the fewer number of lose trials on the MID task meant we were unable to examine voxel-wise group differences in loss anticipation. The end fixation period of the task served as the implicit baseline. Registration was conducted through a two-step procedure, whereby EPI images were first registered to the high-resolution T1 structural image, then into standard (Montreal Neurological Institute, MNI avg152 template) space, with 12-parameter affine transformations.

Higher-level (within group one-sample t-tests and between-group independent samples t-tests) were conducted on the gain anticipation > neutral anticipation contrast using the randomise programme in FSL (Winkler et al., 2014). Randomise employs a permutation approach through resampling. Significance (P_FWE_ < 0.05) for the gain anticipation > neutral anticipation contrast on both the one-sample and independent samples t-tests was conducted taking a threshold-free cluster enhancement (TFCE) approach (Smith and Nichols, 2009) and using 5000 permutations. TFCE aims to maintain the sensitivity of cluster-based thresholding, while avoiding the arbitrary nature of threshold choice. Given the differences in scanners across the three sites, these analyses were conducted while also controlling for study site.

## 7 Trial-Wise beta value image analyses

Data pre-processing was also initially conducted using FEAT (fMRI Expert Analysis Tool) from the FMRIB Software Library (www.fmrib.ox.ac.uk/fsl) as described above. For each participant, each individual gain and neutral anticipation epoch (cue + variable anticipation period) was separately modelled within the context of the general linear model. This approach yielded a total of 18 unique beta value images for each of the gain and neutral anticipation conditions on each run of the MID task. This meant that each voxel-wise beta value image reflected the magnitude of the hemodynamic response evoked by each of the gain and neutral anticipation epochs. Each beta value image for each MID run was then subsequently registered into standard (MNI avg152 template) space before being concatenated to generate a beta value “trial-wise” (e.g., gain anticipation) time series. Each beta value trial-wise time series for each MID run was further concatenated across runs to generate a single beta value trial-wise time series for the gain and neutral MID anticipation conditions. This procedure yielded a thirty-six beta value trial-wise time series image for each participant for the gain and neutral anticipation conditions. The neutral trial-wise time series was then simply subtracted from the gain trial-wise time series to yield a gain anticipation > neutral anticipation contrast time series (see Supplementary Fig. 1). This beta value trial-wise time series method has been previously used to examine connectivity during cognitive tasks (Fornito et al., 2011, Ray et al., 2017), including the MID task (Nestor et al., 2019, Verdejo-Roman et al., 2017). The small, variable (performance-dependent) number of events for the outcome periods (“hits” and “misses”), and the small number of loss trials in the task, meant that we could not generate trial-wise beta value images for these events. Therefore, gain and neutral outcome (well as lose anticipation and outcome) events were regressed out of the functional time series as conditions of no interest during the first level analyses. This meant that the same end fixation period of the task also served as the implicit baseline for these analyses.

## 8 Time series Extraction and correlation matrices

Using the Harvard-Oxford atlas (96 cortical and 14 subcortical nodes/regions) as our connectome of interest, we used the fslmeants programme to extract the mean beta value time series from each of 110 anatomical regions of interest (ROI) for the gain anticipation > neutral contrast beta image for each participant. Using these mean ROI time series outputs, we then conducted Pearson correlation coefficient analyses to construct whole brain ROI‐to‐ROI pairwise matrices (see Supplementary Fig. 1). Each matrix was made up of 5995 (=N*(N − 1)/2, with N = 110 nodes) pairwise connections (edges). These matrices were generated in MATLAB (The MathWorks, Inc., Natick, Massachusetts, United States) and used to compare network connectivity between the CON and ADD groups (see below).

## 9 Graph measures

Global (characteristic path length and clustering coefficient) graph measures were estimated from each correlation matrix using the GraphVar (www.rfmri.org/GraphVar) toolbox for functional brain connectivity (Kruschwitz et al., 2015) in MATLAB (The MathWorks, Inc., Natick, Massachusetts, United States). More detailed descriptions of brain network graph measures can be found elsewhere (Bullmore and Sporns, 2009), but we will briefly describe these metrics here. Characteristic path length is the minimum number of edges that must be traversed to go from one node (brain region) to another in a network. For a pair of nodes that are nearest neighbours, the path length is 1. The clustering coefficient, by contrast, quantifies the density of connections between the nearest neighbour of a node, and describes how segregated the network is. The path and clustering measures are first estimated at each node of the connectome before an average (global value) is computed for the entire connectome for each participant’s correlation matrix.

Graph measures for each participant were then estimated by thresholding each matrix at a selection of proportional cost (*K)* thresholds – i.e. thresholds that retain only a percentage of the strongest connections (edges) in the network. Biological networks are represented by sparse connections (Latora and Marchiori, 2003), however, and thresholding is a necessary step to extract the appropriate topological properties of networks (Achard and Bullmore, 2007). Because graph measures can also be sensitive to threshold value (van Wijk et al., 2010), we have reported our measures across a range of K thresholds (1 ⩽ K ⩽ 5, increments of 1). Here K represents the percentage (e.g., 1 = 10%) number of edges in each matrix that are maintained following thresholding. We employed a range of thresholds to represent the lower and upper bound of a small-world system (Achard and Bullmore, 2007, Bullmore and Bassett, 2011), and that preserve only the strongest functional connections for efficient parallel information processing at a relatively low wiring cost (Latora and Marchiori, 2001). All graph measures were computed from matrices in their weighted form following this thresholding procedure.

## 10 Functional connectivity

Group comparisons in ROI‐to‐ROI connectivity across matrices were assessed using the Networks Based Statistics (NBS) Toolbox (Zalesky et al., 2010) for MATLAB (The MathWorks, Inc., Natick, Massachusetts, United States). Comparisons between the CON and ADD groups were conducted to identify those nodes across the connectome that showed differences in connectivity. Independent groups t-tests were first performed to test for a between-group difference in the correlation coefficients at each of the 110 × (110–1)/2 = 5995 regional pairings. Graph sub-components were identified among the connections using a t-statistic threshold *t* > 3.1. From here, a family-wise error (FWE) corrected *p*-value (*p* < 0.05) was calculated for the size of each resulting component using permutation testing (5000 permutations). Two (CON > ADD and ADD > CON) analyses were conducted independently on the gain anticipation > neutral anticipation contrast matrices. Given the differences in scanners across the three sites, these NBS analyses were conducted while controlling for study site. NBS has previously been used to identify specific networks of nodes across a connectome that differ between clinical populations during different psychological processes (Fornito et al., 2011, Nestor et al., 2019, Ray et al., 2017).

## 11 Network visualisation

Networks that emerged from group comparisons in NBS were visualised and presented using brain connectivity maps and circular connectograms using the NeuroMArVL software (www.immersive.erc.monash.edu.au/neuromarvl).

## 12 Results

### 12.1 Demographics

Table 1 (supplementary section) shows the demographic and substance use measures for the CON and ADD groups. The groups were balanced for gender and age, but did significantly differ on other measures, such as reported education, IQ, anxiety, depression, MINI score, and the number of participants who were nicotine-dependent. While we cannot dismiss the potential influence of these demographic differences on brain activation changes or connectivity during reward anticipation, we did not use any of these variables as covariates in any of the analyses reported.

## 13 MID task

**Fig. 1:**
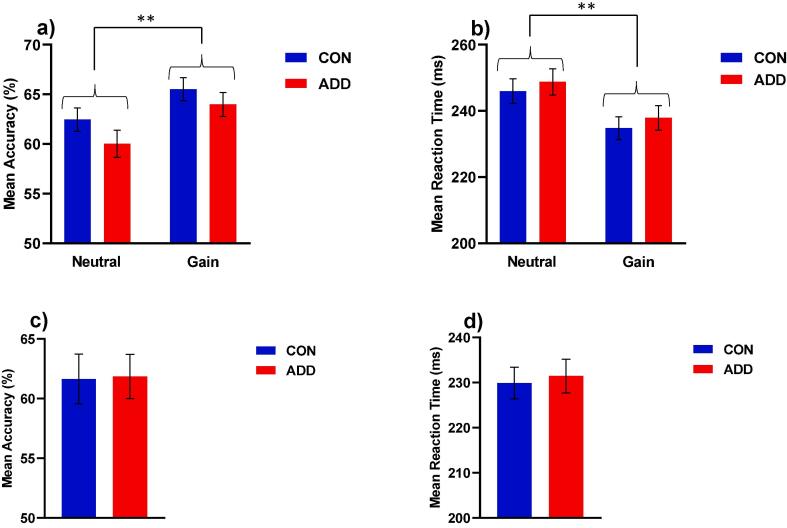
MID performance results for the CON and ADD group showing a) mean accuracy (^**^p < 0.01 Gain > Neutral); b) mean reaction time (^**^p < 0.01 Gain < Neutral); c) mean lose accuracy and d) mean lose reaction time. Data were analysed with permutation analyses of variance (5000 permutations), controlling for study site. Data are expressed as means and standard error means.

Two (Group: CON vs. ADD) by two (Condition: Gain vs. Neutral) permutation analyses of variance showed only a significant effect of condition for MID accuracy (*F* = 7.8, *p* < 0.01 - Gain > Neutral) and MID reaction time (*F* = 8.49, *p* < 0.01 - Gain < Neutral - Fig. 1a and b). Permutation analyses did not reveal any significant effect of group on the MID lose accuracy (*F* = 0.01, *p* = 0.94) or reaction time (*F* = 0.09, *p* = 0.77) measures (Fig. 1c and d), or the amount won (£) on the task (*F* = 0.45, *p* = 0.50). These results suggest that the CON and ADD groups were well matched with respect to MID performance, while also validating the task with respect to its effects on instrumental responding for monetary reward.

## 14 fMRI

**Fig. 2:**
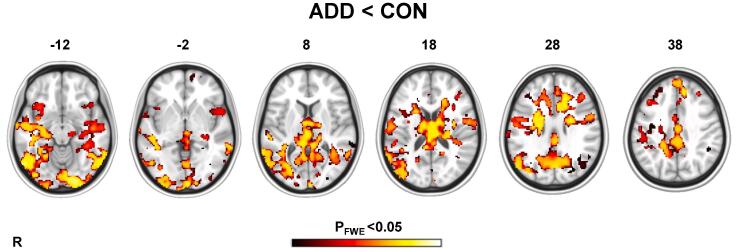
Permutation independent samples *t*-test analyses showing that ADD < CON group activation differences on the MID gain anticipation > neutral anticipation contrast. Images were produced after 5000 permutations in randomise using TFCE (P_FWE_ < 0.05), controlling for study site. The bar corresponds to P_FWE_ < 0.05 and lower. The structural image represents the MNI152 average normal brain with corresponding horizontal coordinates (inferior–superior). R = right hemisphere.

One-sample permutation *t*-test analyses showed that both the CON and ADD groups activated a mostly frontostriatal network of regions for gain anticipation > neutral anticipation contrast (TFCE, P_FWE_ < 0.05) – although these activation changes were weaker in the ADD group (see Supplementary Fig. 2). Independent samples permutation *t*-test analyses on the same contrast detected significant differences between the two groups. The ADD group showed significantly less activation change compared with the CON group (TFCE, P_FWE_ < 0.05), particularly across temporal (including the amygdala, hippocampus) and visual regions. There were also less pronounced differences across frontal (insula, inferior frontal gyrus) and limbic-associated (anterior cingulate gyrus, thalamus) regions (Fig. 2). These between groups analyses, however, did not reveal any differences in activation change across striatal regions.

## 15 Graph measures

**Fig. 3:**
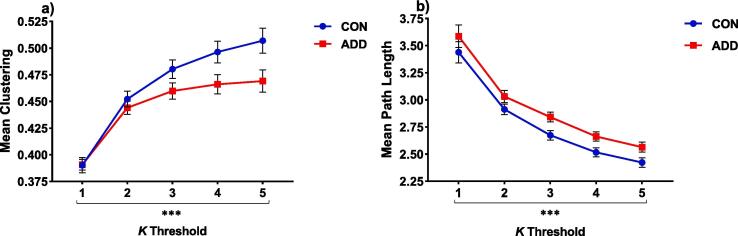
Global network differences between groups showing a) clustering (***p < 0.001, ADD < CON) and b) path length (**p < 0.01, ADD > CON) during the gain > neutral contrast of the MID task. Data were analysed using two (group: CON vs. ADD) × five (1 ⩽ *K* ⩽ 5) permutation anova analyses, controlling for study site. Data are expressed as means and standard error means.

The results from all permutation tests are provided in the Supplementary results section. Here we report only the main effects of Group. Two (Group: CON vs. ADD) by five (1 ⩽ K ⩽ 5) permutation analyses of variance showed a significant effect of Group on both the clustering coefficient *(F =* 29.98*, p <* 0.001 - ADD < CON) and characteristic path length (*F =* 7.30, *p <* 0.001 – ADD > CON) measures (Fig. 3a and b).

## 16 Functional connectivity

**Fig. 4:**
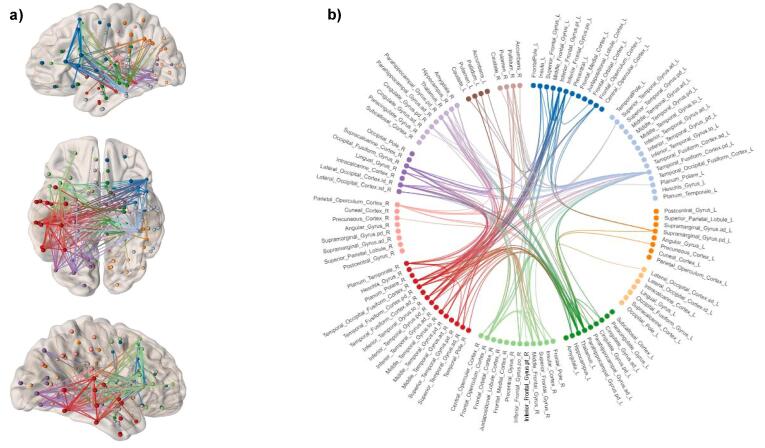
Non-parametric network based statistics (NBS) analysis results showing a graph sub-component comprising 153 edges (*p* < 0.01) where the ADD group demonstrated significantly less connectivity compared to the CON group during the gain > neutral anticipation contrast of the MID task. Graph sub-components were identified among all node pairwise connections with a *t*-statistic threshold of *t* > 3.1, corrected for multiple comparisons, while controlling for study site using permutation (5000) analyses. Reductions in connectivity in the ADD group are represented by a) brain connectivity maps and b) a circular connectogram. Brain regions are grouped on the connectogram circumference according to lobes and centres in the left and right hemispheres (left frontal [dark blue]; left temporal [light blue]; left parietal [dark orange], left occipital [light orange]); left limbic [dark green]; right frontal [light green]; right temporal [dark red]; right parietal [pink]; right occipital [dark purple]; right limbic [light purple]; left striatal [dark brown] and right striatal [light brown]. Brain connectivity maps and the circular connectogram were generated using NeuroMArVL (http://immersive.erc.monash.edu.au/neuromarvl). (For interpretation of the references to colour in this figure legend, the reader is referred to the web version of this article.)

The network based statistics (NBS) analyses detected a graph sub-network comprising 153 edges between 59 nodes of the connectome where the ADD group demonstrated significantly less connectivity (*p* < 0.01) compared with the CON group. These differences in connectivity were mostly intra-hemispheric (55%), the majority (38%) being in the right hemisphere. The anatomical distribution of these connectivity differences between the two groups involved frontal (insula, inferior frontal gyrus, orbitofrontal cortex), limbic-associated (anterior cingulate gyrus, thalamus), visual (lateral occipital cortex, lingual gyrus, intracalcarine cortex); and unlike the voxel-wise analyses, striatal (accumbens, caudate, pallidum) regions (Fig. 4).

## 17 Discussion

We compared a control (CON) and an addiction (ADD) group in extended abstinence on behavioural and neural measures of a Monetary Incentive Delay (MID) task. Behaviourally, the two groups were well matched, enabling us to discount performance as a potential confound on group differences in brain activation and connectivity. These performance effects showed that both groups were equally incentivized to maximize monetary gains, as revealed by significant differences between the gain and neutral conditions. These results, therefore, validate the incentivizing effects of our MID task on instrumental responding for rewards. The two groups did, however, show significant differences in brain activation, and to a greater degree, global network connectivity related to the anticipation of monetary reward. These differences, particularly in global connectivity, point to the preservation of widespread neural disturbances in an addiction population, despite being in extended abstinence.

## 18 Disruptions to whole brain activation in the ADD group

Here we report that the ADD group showed significantly less activation change compared with the CON group, most robustly across temporal and visual cortical regions during the anticipation of monetary reward. Addiction disorders are commonly associated with disturbances in the reward network (Balodis and Potenza, 2015, Hommer et al., 2011, Just et al., 2019, Koob and Le Moal, 2005, Luijten et al., 2017, Wrase et al., 2007), and the present results did also show some evidence for less activation change across reward-associated regions, the most pronounced of which were in the anterior cingulate gyrus (ACG) and amygdala. Disturbances in ACG functioning have been commonly reported in addiction populations (Goldstein and Volkow, 2002, Peoples, 2002, Volkow et al., 2002), which persist into abstinence (Bolla et al., 2004, Eldreth et al., 2004, Nestor et al., 2011, Nestor et al., 2017, Salloum et al., 2007). The differences observed in the current sample were within the dorsal ACG, a region implicated in motivation and cognitive control (Kouneiher et al., 2009). The amygdala also encodes stimulus value (Jenison et al., 2011), with disruptions also evident in addiction (Lesscher and Vanderschuren, 2012, See et al., 2003). These results point to localised neural disturbances in regions implicated in motivational, executive functioning and reward evaluation, that endure into extended abstinence.

This current set of results, however, was in the absence of group differences in striatal activation between the ADD and CON groups. This does not appear to concur with previous research findings of altered striatal responses for non-drug rewards in substance dependence (Buhler et al., 2010, Bustamante et al., 2014, Diekhof et al., 2008, Gradin et al., 2014, Murphy et al., 2017, Nestor et al., 2017, Peters et al., 2011, Wrase et al., 2007), and does not demonstrate evidence of a sustained striatal “reward deficiency syndrome” (Blum et al., 2000, Koob et al., 2004) in this particular sample. The heterogenous nature of the ADD sample with respect to mono- and multiple- drug dependencies and the variable abstinence length from these substances, may have been a constraining factor, reflecting varying levels of restoration of function in the striatum (and other regions). For example, studies have shown there are deficits in markers of striatal dopamine functioning during early abstinence (Boileau et al., 2016, Martinez et al., 2005, Wang et al., 2012), with the variable abstinence length in the current sample masking possible disturbances in this region. Another possibility is that a voxel-wise approach was insufficiently sensitive to detect group differences across smaller regions such as the striatum. The results from the network analyses (discussed below) did reveal striatal regions (e.g., accumbens, caudate) that were part of a sub-network of nodes demonstrating less connectivity in the ADD group. This may further endorse the sensitivity of exploring measures of functional network connectivity over voxel-wise approaches for detecting more extensive disruptions in neural processing. Finally, it is possible that the ADD and CON groups simply did not differ in the striatum, which has recently been shown in other studies of stimulant users (e.g. Just et al., 2019).

## 19 Disruptions in global network connectivity in the ADD group

The ADD group had an increased mean global characteristic path length compared to the CON group. As described above, path length is a topological measure indicating the average shortest path length between all node pairs in a network, whereby fewer processing steps across the network has the advantage of propagating more rapid and accurate communication (Kaiser and Hilgetag, 2006). We observed this metric to be elevated in the ADD group, representing a possible reduction in processing efficiency. This may be due to a loss of long range connections between remote brain regions, which are critical for minimising path lengths and maintaining network efficiency. This disruption in processing efficiency concurs with that reported in other addiction populations during rest (Holla et al., 2017, Lin et al., 2015, Morris et al., 2018, Wang et al., 2015), but not during reward processing (Nestor et al., 2019).

We further report that the ADD group demonstrated a lower clustering coefficient across the network. Clustering quantifies the number of connections existing between a node’s nearest neighbours, which has been proposed as an index of local specialised processing and economic pressure for minimal wiring cost (Bullmore and Bassett, 2011, Kaiser and Hilgetag, 2006, Rubinov and Sporns, 2010, Sporns et al., 2004). The reduction in clustering we report here contrasts with the increased clustering found in an addiction population during resting state, and which was reversed by acute naltrexone (Morris et al., 2018). Alterations in clustering observed during reward anticipation may suggest less interconnectedness in local networks for specialised or segregated information processing, which could be more economically costly for brain functioning (Bullmore and Sporns, 2012). Significantly, these global functional alterations could be due to changes in anatomical network architecture, as functional connectivity is generally tightly aligned with anatomical connectivity (Honey et al., 2009, Sporns, 2011, van den Heuvel et al., 2009).

## 20 Decreased reward-related connectivity in the ADD group

Taking a network based statistics (NBS) analysis approach, we showed that the ADD group exhibited reduced connectivity across a sub-network of nodes during the anticipation of reward. This reduction in connectivity comprised a total of 153 connections, the majority of which were confined to the right hemisphere. The anatomical distribution of these between-group connectivity differences involved frontal (insula, inferior frontal gyrus, orbitofrontal cortex), striatal (accumbens, caudate), limbic-associated (ACG, amygdala, hippocampus) and visual (lateral occipital cortex, lingual gyrus, intracalcarine cortex) regions. Similar analysis approaches have also revealed differences across sub-networks in addiction, however, during resting state (Hong et al., 2013, Morris et al., 2018, Wee et al., 2014). The connectivity differences reported in this ADD sample indicate alterations between cognitive, striatal and limbic-associated regions during reward anticipation that persist into extended abstinence. Furthermore, the emergence of a more extensive network of regions in this connectivity analysis highlights a more sensitive approach for detecting widespread disruptions across neural networks in addiction, that may fail to emerge through conventional voxel-wise analysis approaches.

The presence of these connectivity differences across a distributed sub-network of nodes, concomitant with differences on global topological measures, is also noteworthy. Previous studies using combined graph- and network-based approaches have failed to report differences using both types of measures (Cocchi et al., 2012, Fornito et al., 2011, Hong et al., 2013). While topological and network-based measures of connectivity may be distinct, there does appear to be some convergence across both analyses that suggests the preservation of functional disruptions in a latent and more widespread network in this addiction group.

## 21 Conclusion

The spatial distribution of functional disturbances reported in addiction populations during functional MRI studies is commonplace, and is likely driven by extensive disruptions in connectivity across the whole brain network. The current study, taking a novel analytical approach, has revealed reward-related functional alterations across a global network in an addiction disorder population who are in extended abstinence. These differences in connectivity feature in the presence of more localised activation disruptions that emerge from a conventional voxel-wise analytic approach. We propose that examining measures of global network functioning may be more sensitive for highlighting latent and more widespread disruptions to neural functioning during critical psychological processes in addiction and other psychiatric disorders. Alterations in functioning across global networks could act as markers for relapse risk during abstinence, that may be potential targets for medication development in addiction disorders.

## Funding

The authors disclosed receipt of the following financial support for the research, authorship, and/or publication of this article: This article presents independent research funded by the MRC as part of their addiction initiative (grant number G1000018). GSK kindly funded the functional and structural MRI scans that took place at the London site for this study.

## Declaration of competing interests

The authors declared the following potential conflicts of interest with respect to the research, authorship, and/or publication of this article:

David J Nutt is an advisor to British National Formulary, MRC, General Medical Council, Department of Health, is President of the European Brain Council, past President of the British Neuroscience Association and European College of Neuropsychopharmacology, chair of the Independent Scientific Committee on Drugs (UK), is a member of the International Centre for Science in Drug Policy, advisor to Swedish government on drug, alcohol and tobacco research, editor of the Journal of Psychopharmacology, sits on advisory Boards at Lundbeck, MSD, Nalpharm, Orexigen, Shire, has received speaking honoraria (in addition to above) from BMS/Otsuka, GSK, Lilly, Janssen, Servier, is a member of the Lundbeck International Neuroscience Foundation, has received grants or clinical trial payments from P1vital, MRC, NHS, Lundbeck, has share options with P1vital, has been expert witness in a number of legal cases relating to psychotropic drugs, and has edited/written 27 books, some purchased by pharmaceutical companies.

Trevor W Robbins has research grants with Eli Lilly and Lundbeck, has received royalties from Cambridge Cognition, has received editorial honoraria from Springer Verlag, Elsevier, Society for Neuroscience; has performed educational lectures for Merck, Sharpe and Dohme and does consultancy work for Cambridge Cognition, Eli Lilly, Lundbeck, Teva and Shire Pharmaceuticals.

Barbara J Sahakian consults for Cambridge Cognition, Greenfield BioVentures, and Casssava Sciences.

William Deakin currently advises or carries out research funded by Autifony, Sunovion, Lundbeck, AstraZeneca and Servier. All payment is to the University of Manchester.

Edward T Bullmore was employed half-time by the University of Cambridge and half-time by GSK during some of this work and is a shareholder in GSK.

Liam J Nestor was employed by GSK during some of this work.

Eugenii Rabiner worked for GSK until 2011 and is a shareholder in GSK. He is a consultant to GSK, TEVA, Lightlake Therapeutics, AbbVie, and Roche.

## CRediT authorship contribution statement

**Liam J. Nestor:** Conceptualization, Methodology, Software, Validation, Formal analysis, Data curation, Writing - original draft, Visualization. **John Suckling:** Conceptualization, Resources, Writing - original draft, Supervision, Project administration. **Karen D. Ersche:** Investigation, Supervision, Funding acquisition, Project administration, Writing - original draft. **Anna Murphy:** Investigation, Data curation, Project administration. **John McGonigle:** Investigation, Data curation, Project administration. **Csaba Orban:** Investigation, Data curation, Project administration. **Louise M. Paterson:** Investigation, Data curation, Project administration, Writing - original draft. **Laurence Reed:** Funding acquisition. **Eleanor Taylor:** Investigation, Data curation, Project administration. **Remy Flechais:** Investigation, Data curation, Project administration. **Dana Smith:** Investigation, Data curation, Project administration. **Edward T. Bullmore:** Funding acquisition. **Rebecca Elliott:** Funding acquisition. **Bill Deakin:** Funding acquisition. **Ilan Rabiner:** Funding acquisition. **Anne-Lingford Hughes:** Writing - original draft, Funding acquisition. **Barbara J. Sahakian:** Writing - original draft, Funding acquisition. **Trevor W. Robbins:** Writing - original draft, Supervision, Funding acquisition. **David J. Nutt:** Writing - original draft, Supervision, Funding acquisition. **:** .
