# Supplementary material for: Disturbances across whole brain networks during reward anticipation in an abstinent addiction population
Source: Neuroimage Clin. 2020 May 26;27:102297. doi: 10.1016/j.nicl.2020.102297 (PMC7270610; doi:10.1016/j.nicl.2020.102297)
Supplement: Supplementary data 1 [file mmc1.docx]

**Supplementary Figure 1**. Pipeline showing 1) generation of the beta value time series images for the gain, neutral and gain minus neutral anticipation conditions in FSL, 2) extraction of beta value time series from nodes of the Harvard-Oxford structural atlas using FSLMEANTS, 3) construction of the correlation matrices from the beta value time series in MATLAB, 4) generation of graph theory measures, and 5) between group analyses of graph theory measures and network-based statistics (NBS).
