# Supplementary material for: Disturbances across whole brain networks during reward anticipation in an abstinent addiction population
Source: Neuroimage Clin. 2020 May 26;27:102297. doi: 10.1016/j.nicl.2020.102297 (PMC7270610; doi:10.1016/j.nicl.2020.102297)
Supplement: Supplementary data 2 [file mmc2.docx]

**Supplementary Figure 2**. Permutation one-sample t-test analyses showing the mean activation in a) the CON group and b) the ADD group for the MID gain anticipation>neutral anticipation contrast. Images were produced after 5000 permutations in randomise using TFCE (P_FWE_<0.05 ), controlling for study site. The bar corresponds to P_FWE_<0.05 and lower. The structural image represents the MNI152 average normal brain with corresponding horizontal coordinates (inferior–superior). R=right hemisphere.
