# Supplementary material for: Disturbances across whole brain networks during reward anticipation in an abstinent addiction population
Source: Neuroimage Clin. 2020 May 26;27:102297. doi: 10.1016/j.nicl.2020.102297 (PMC7270610; doi:10.1016/j.nicl.2020.102297)
Supplement: Supplementary data 3 [file mmc3.docx]

**Supplementary Table 1.** Showing the demographic, questionnaire and drug use measures for the control (CON) and addiction (ADD) groups. The groups differed significantly on a number of the measures, including education (****p*<0.001, ADD<CON); IQ (****p*<0.001, ADD<CON); total BDI score (****p*<0.001, ADD>CON); total STAI-T score (****p*<0.001, ADD>CON); total MINI score (****p*<0.001, ADD>CON) and nicotine dependence (****p*<0.001, ADD>CON). Data were analysed using permutation independent sample t-test analyses. Data are expressed as means and standard error means.

|  |  |  |  |
| --- | --- | --- | --- |
|  |  | **CON (n=68)** | **ADD (n=83)** |
|  |  |  |  |
| **Gender (female/male)** |  | 18/50 | 16/67 |
|  |  |  |  |
| **Age** |  | 39.79 ± 1.22 | 40.50 ± 0.92 |
|  |  |  |  |
| **Education (years)** |  | 14.14 ± 0.32 | 11.61 ± 0.26*** |
|  |  |  |  |
| **WTAR IQ Score** |  | 108.97 ± 1.04 | 100.75 ± 1.11*** |
|  |  |  |  |
| **Handedness Score** |  | 51.45 ± 7.50 | 65.46 ± 6.18 |
|  |  |  |  |
| **BDI Score (total)** |  | 3.20 ± 0.47 | 9.22 ± 0.85*** |
|  |  |  |  |
| **STAI-T Score (total)** |  | 29.94 ± 0.95 | 40.06 ± 1.27*** |
| **MINI Score (total)** |  | 0.79 ± 0.22 | 4.22 ± 0.46*** |
|  |  |  |  |
| **Dependencies (%)** |  |  |  |
| *nicotine* |  | 52 | 87*** |
| *alcohol* |  | - | 72 |
| *cocaine* |  | - | 51 |
| *opiates* |  | - | 44 |
| *amphetamine* |  | - | 10 |
| *Other*^✝^ |  | - | 16 |
|  |  |  |  |
|  |  |  |  |

^✝^ benzodiazepines; ketamine, Gamma Hydroxybutyrate; inhalants

Wechsler Test of Adult Reading (WTAR)

Beck Depression Inventory (BDI) Questionnaire

State Trait Anxiety Inventory (STAI) Questionnaire

Mini-International Neuropsychiatric Interview (MINI)
