# Supplementary material for: Disturbances across whole brain networks during reward anticipation in an abstinent addiction population
Source: Neuroimage Clin. 2020 May 26;27:102297. doi: 10.1016/j.nicl.2020.102297 (PMC7270610; doi:10.1016/j.nicl.2020.102297)
Supplement: Supplementary data 4 [file mmc4.docx]

Supplementary graph results for Gain Anticipation>Neutral Anticipation Contrast

The Permutation Tests for Linear Models (“lPerm”) programme in the R analysis package was used to conduct the two (Group: CON vs. ADD) by five (1 ⩽ K ⩽ 5) analyses of variance. All follow-up comparisons between the *K* thresholds were conducted using pairwise testing, which in the current set of analyses, used a Bonferroni correction for multiple comparisons procedure in the R analysis package.

For the clustering coefficient, a two (Group: CON vs. ADD) by five (1 ⩽ K ⩽ 5) permutation analysis of variance showed a significant effect of group (*F=*29.98*, p<*0.001 - ADD<CON), *K* threshold (*F=* 397.92*, p<*0.001 – 10%<20%, 30%, 40%, 50%, *p<*0.001_Bonferroni_; 20%<30%, 40%, 50%, *p<*0.001_Bonferroni_; 30%<40%, *p<*0.05_Bonferroni_; 30%<50%, *p<*0.001_Bonferroni_), but no group x *K* threshold interaction (*F=*0.59*, p=*0.44).

For characteristic path length, a two (Group: CON vs. ADD) by five (1 ⩽ K ⩽ 5) permutation analysis of variance showed a significant effect of group (*F=*7.30, *p<*0.001 - ADD>CON), *K* threshold (*F=* 288.82, *p<*0.001 - 10%<20%, 30%, 40%, 50%, *p<*0.001_Bonferroni_; 20%<30%, *p*<0.01_Bonferroni_; 20%<30%, 50%, *p*<0.001_Bonferroni_; 30%<50%, *p*<0.001_Bonferroni_), but no group x *K* threshold interaction (*F=*0.054, *p=*0.81).
